# CHRM3 is a novel prognostic factor of poor prognosis and promotes glioblastoma progression via activation of oncogenic invasive growth factors

**DOI:** 10.32604/or.2023.030425

**Published:** 2023-09-15

**Authors:** BIN ZHANG, JIANYI ZHAO, YONGZHI WANG, HUA XU, BO GAO, GUANGNING ZHANG, BIN HAN, GUOHONG SONG, JUNCHEN ZHANG, WEI MENG

**Affiliations:** 1 Department of Neurosurgery, Affiliated Hospital of Jining Medical University, Jining, China; 2 Department of Neurosurgery, The City Peoples’ Hospital of Fuyang, Fuyang, China; 3 Brain Injury Center, Department of Neurosurgery, Ren Ji Hospital, Shanghai Jiao Tong University School of Medicine, Shanghai, China

**Keywords:** Glioblastoma, Prognosis, CHRM3, MMP, CXCL

## Abstract

Glioblastoma (GBM) is the most aggressive cancer of the brain and has a high mortality rate due to the lack of effective treatment strategy. Clarification of molecular mechanisms of GBM’s characteristic invasive growth is urgently needed to improve the poor prognosis. Single-nuclear sequencing of primary and recurrent GBM samples revealed that levels of M3 muscarinic acetylcholine receptor (CHRM3) were significantly higher in the recurrent samples than in the primary samples. Moreover, immunohistochemical staining of an array of GBM samples showed that high levels of CHRM3 correlated with poor prognosis, consistent with The Cancer Genome Atlas database. Knockdown of CHRM3 inhibited GBM cell growth and invasion. An assay of orthotopic GBM animal model *in vivo* indicated that inhibition of CHRM3 significantly suppressed GBM progression with prolonged survival time. Transcriptome analysis revealed that CHRM3 knockdown significantly reduced an array of classic factors involved in cancer invasive growth, including MMP1/MMP3/MMP10/MMP12 and CXCL1/CXCL5/CXCL8. Taken together, CHRM3 is a novel and vital factor of GBM progression via regulation of multiple oncogenic genes and may serve as a new biomarker for prognosis and therapy of GBM patients.

## Introduction

Glioblastoma (GBM), with great growth and invasion abilities, is the most universal and lethal tumor in the central nervous system [1–4]. The median survival time of patients suffered GBM is often less than 15 months, although great efforts have been made in the treatment of GBM patients [5–7]. Thus, it is urgently needed to explore GBM biology and determine new molecular targets that are beneficial for therapeutic effect.

A growing body of research has clarified multiple molecules that are crucial for GBM malignant progression. Among them, the upregulation of matrix metalloproteinases (MMPs) and chemokine (C-X-C motif) ligand family (CXCL) expression plays a significant role in promoting GBM pathogenesis [8–11]. In particular, elevated levels of MMP1 are an indicator of GBM invasion and are essential for guanylate binding protein 5 (GBP5)-promoted GBM aggressiveness [12,13]. An increase in CXCL5 is also found in GBM and indicates poor prognosis [14]. CXCL1 regulates radio-resistance of GBM [15]. CXCL8 promotes GBM aggravation through the JAK/STAT1/HIF-1α/Snail signaling pathway [16].

The expression of muscarinic receptors, mainly the M3 subtype (CHRM3), is amplified in several types of tumors, including colon cancer [17], prostate cancer [18], endometrial carcinoma [19], and gastric cancer [20]. Colon cancer overexpresses CHRM3, and post-M3R signaling promotes cell growth via the activation of the epidermal growth factor receptors/ERK and protein kinase C/p38 mitogen-activated protein kinase (MAPK) signaling pathways [21]. In contrast, MMP1 production and colon carcinoma invasion may be weakened via CHRM3 associated signals suppression [21]. Recently, several scoring systems, such as 16-gene signature scoring system and 9-gene signature scoring system, have emerged to evaluate the prognosis of GBM, and all of them include CHRM3 [22]. In addition, dysregulated genes that encode neurotransmitter receptors are considered to have differential associations with patient survival in GBM and low-grade glioma [23]. However, the pathological functions and mechanisms of CHRM3 in GBM remain unknown [24].

In the present study, we collected six fresh samples of primary and recurrent GBM and analyzed them using single-nuclear sequencing. The Levels of CHRM3 were notably higher in the recurrent samples than in the primary samples. Moreover, the expression levels of CHRM3 negatively correlated with the prognosis of GBM patients not only in The Cancer Genome Atlas (TCGA) database but also in an array of GBM samples. Functional studies *in vitro* and *in vivo* indicated that CHRM3 was crucial for GBM invasive growth. Transcriptome analysis revealed that CHRM3 knockdown significantly reduced an array of classic factors in cancer invasive growth including MMP1/MMP3/MMP10/MMP12 and CXCL1/CXCL5/CXCL8. Thus, our study identified a novel and vital factor of GBM development and CHRM3 may be a new target for GBM prognosis and therapy.

## Materials and Methods

### Patients’ tissue samples

The Ethics Committee of Renji Hospital, School of Medicine, Shanghai Jiao Tong University permitted this study (IRB number, RA-2022-032). Consent was acquired from patients and all studies were performed in accordance with relevant regulations. Three primary and three recurrent fresh samples were collected for single‑nuclear sequencing. And sixty-five paraffin-embedded samples of patients with primary GBM and their follow-up data at Renji Hospital, Shanghai Jiao Tong University between January 2005 and December 2019 were included. The age at diagnosis of the patients with GBM was from 22 to 76 years, with 36 males and 29 females. The primary and recurrent samples were not collected from the same patient. And four fresh normal tissues were collected from patients with traumatic brain injury.

### Single‑nuclear sequencing library preparation and data processing

Single-nuclear RNA sequencing was carried out on the fresh samples collected from patients with GBM treated at Ren Ji Hospital, Shanghai Jiao Tong University. The samples were stored for research purposes in line with an IRB approved protocol. The manufacturer’s protocol of Chromium Next GEM Single Cell 3′ Reagent Kits v3.1 was followed when preparing the libraries. The Cell Ranger software pipeline (version 3.1.0) provided by 10 × Genomics was used to demultiplex cellular barcodes, map reads to the genome and transcriptome using the STAR aligner, and down-sample reads as required to generate normalized aggregate data across the samples, producing a matrix of gene counts *vs*. cells. R package Seurat (version 3.1.1) was used to process the unique molecular identifier count matrix. The sequencing and bioinformatics analysis were performed by Oebiotech Co., Ltd. (Shanghai, China).

### Survival analysis from TCGA database

Gene levels and related pathological information of GBM were taken from TCGA. Gene expression levels were classified as distributed into high- or low-expression. Survival estimates were obtained through Kaplan–Meier analysis and the survival differences were analyzed using the Mantel–Haenszel test.

### Western blot

Western blot (WB) assays were done with rabbit anti-human CHRM3 (cat Ab126168, Abcam, Cambridge, UK) and mouse anti-actin antibodies (cat mAbcam 8226, Abcam, Cambridge, UK). In brief, the samples were lysed with RIPA buffer (cat R0010, Solarbio, Beijing, China) containing protease inhibitors (SKU 11836153001, Roche, Basel, Switzerland). The lysates were divided on 10% SDS-PAGE gels (cat 20325ES62, Yeason, Shanghai, China). The membranes (cat GVWP02500, Millipore, MA, USA) were incubated with rabbit anti-human CHRM3 antibody (1:1000) or mouse anti-actin antibody (1:10,000). HRP-conjugated secondary antibodies were used, and bands were spotted using an ECL kit (cat PI32209, Thermo Scientific Pierce, Waltham, MA, USA).

### qRT-PC

RNA was extracted using TRIzol® reagent (cat 15596026CN, Thermo Fisher Scientific, Waltham, MA, USA). Reverse transcription of RNA was done using HiScript II RT SuperMix (cat R223-01, Vazyme, Nanjing, China). Forward primer for CHRM3 was 5′-CGTGGCACCTGGTCTCTTTC-3′ and reverse primer for CHRM3 was 5′-TTCCAGGTAGGAGCATCAAACC-3′.

### Cells

Four human GBM cell lines (U87-MG, A172, U251-MG and T98G) were acquired from Shanghai Institute of Cell Biology, Chinese Academy of Sciences (Shanghai, China), and cultivated in modified Eagle’s medium (cat SH30022.02, HyClone, Logan, UT, USA) complemented with fetal bovine serum (10%) (cat 10100147, Gibco, Carlsbad, CA, USA), penicillin (100 units/mL) and streptomycin (100 μg/mL) at 37°C with 5% CO_2_.

### Lentivirus, plasmid construction and transfection

CHRM3 knockdown (KD) or overexpression (OE) lentivirus was produced by Hanyin Biotech (Shanghai, China). Among them, U87-MG and T98G cells were infected with CHRM3-KD lentivirus while A172 and U251-MG cells were infected with CHRM3-OE lentivirus. CHRM3-KD sequences were as follows: KD1, 5′-CCTGTGCCGATCTGATTAT-3′; KD2, 5′-GAGGATCTATAAGGAAACT-3′; KD3, 5′-GTGGTCTTCATCGCTTTCT-3′.

### Cell proliferation assay

The cells were planted at 2500 cells/well in a 96-well plate. A total of 10 μL of CCK-8 reagent (cat CK04-01, Dojindo, Kumamoto, Japan) was applied. Absorbance at OD450 was collected at 24, 48, 72, 96 and 120 h. Test was done at least in triplicate.

### Colony formation experiments

A total of 100 cells/well were planted in plates (6-well). After 9 days, the formed colonies were fixed and marked with crystal violet (0.1%). The number of colonies (>200 cells/colony) was calculated. Each experiment was done in triplicate.

### Matrigel-transwell experiments

The cells were seeded in the top chamber coated with Matrigel (cat CLS3422, 8-µm pores, Millipore, MA, US). The bottom chamber was filled with complete medium. The top layer was cleaned with a sterilized cotton swab to eliminate residual cells after overnight incubation. The migrated cells were stained with 0.1% crystal violet and counted. The number of cells was calculated in five random fields per well, and the average number was noted. The assays were conducted in triplicate.

### Wound healing assays

The cells (90% confluence) were seeded in plates (6-well) and rubbed with a 200 µL pipette tip. Healing ability was calculated as the percentage change of the initial induced injury width. Each experiment was done in triplicate.

### Xenograft animal model

The animal study was permitted by the Institutional Animal Care and Use Committee of Renji Hospital, Shanghai Jiao Tong University (IRB number, B-2019-003). Athymic male nu/nu mice (Lingchang Biotech, Shanghai, China) were used to build the GBM model. U87-MG control (5 × 10^5^/5 μL) and CHRM3 knockdown cells (5 × 10^5^/5 μL) were embedded into the *corpus* striatum of anaesthetized athymic nude mice (6 weeks old) via a stereotactic frame (David Kopf Instruments). The mice were monitored daily and examined with MRI scanning when they presented weight loss or neurologic impairments. Tumor diameters were measured in the MRI images and tumor volume was calculated as (length × width^2^)/2 using Function Analysis software (General Electric). Survival time of tumor-burden mice was checked. The mice were euthanized when they were severely emaciated and depressed. The mice were operated on and housed according to the criteria outlined in the Guide for the Care and Use of Laboratory Animals. All of the experiments were approved by the Institutional Animal Care and Use Committee of Shanghai Jiao Tong University, School of Medicine. The mice were randomly assigned to groups.

### Transcriptome assay

Transcriptome assay was carried out by Meiji Biotech (Shanghai, China). Briefly, total RNA was extracted from the tissue using TRIzol® Reagent according to the manufacturer’s instructions (Invitrogen), and genomic DNA was removed using DNase I (TaKara). Then, RNA quality was determined using 2100 Bioanalyser (Agilent), and RNA was quantified using the ND-2000 (NanoDrop Technologies). RNA-sequencing transcriptome library was prepared using TruSeqTM RNA sample preparation kit from Illumina (San Diego, CA, USA) with 1 μg of total RNA. After quantification by TBS380, paired-end RNA sequencing library was sequenced with the Illumina HiSeq xten/NovaSeq 6000 sequencer (2 × 150 bp read length).

### Statistical analysis

Survival curve of CHRM3 was analyzed using the Kaplan-Meier method and log-rank test. The medium survival time and hazard ratio were revealed as 95% confidence interval. Student’s *t*-test (two-tailed) was used for the analysis of the difference between the two groups. The statistical analyses were conducted in SPSS version 17.0 (Chicago, IL, USA). The results were treated as significant when *p* value was below 0.05 (two-sided).

## Results

### CHRM3 is upregulated in GBM and predicts poor prognosis

**FIGURE 1:**
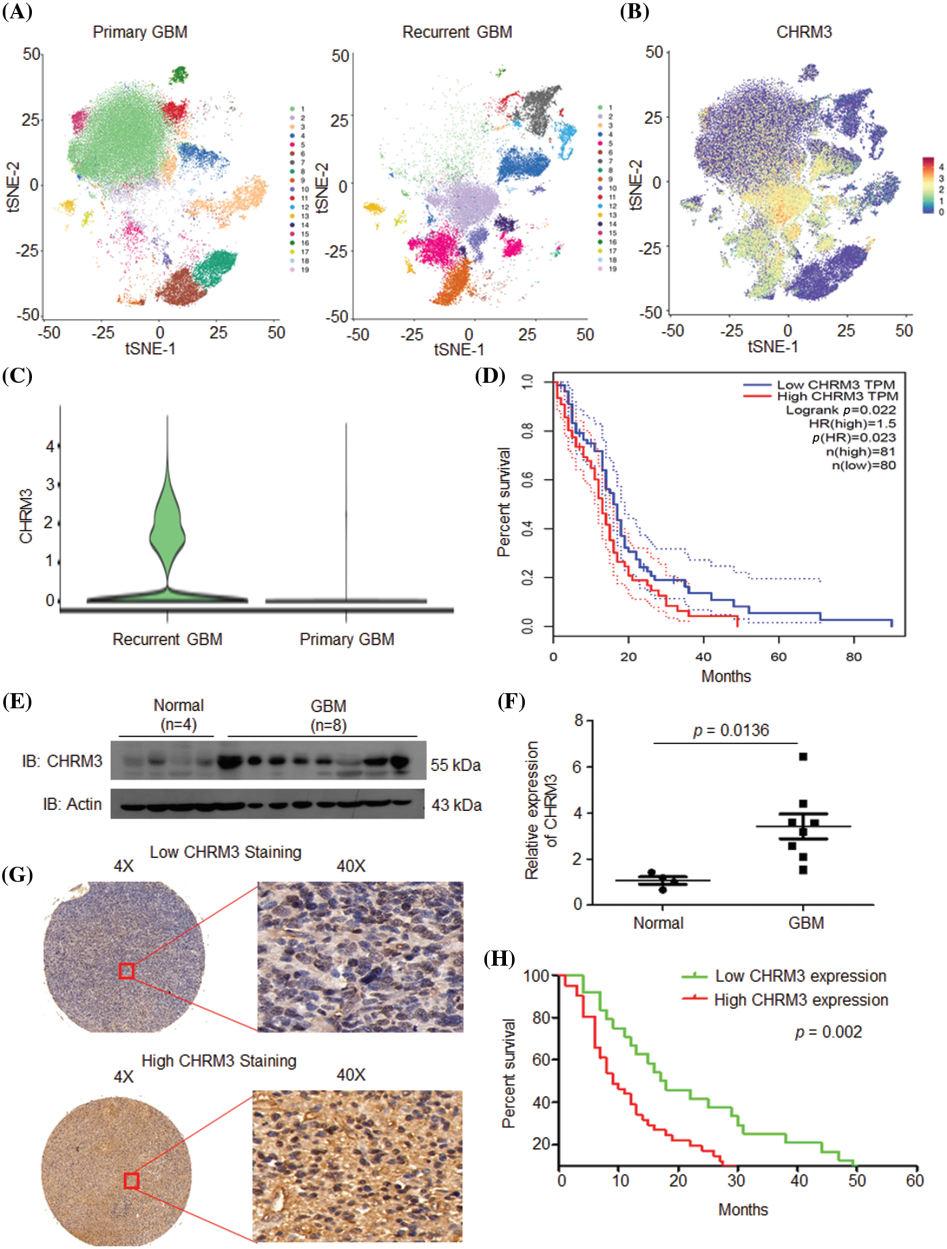
CHRM3 was highly expressed in GBM and predicted poor prognosis. (A) tSEN dimensional reduction of GBM cells from primary (n = 3) or recurrent (n = 3) GBM patients. (B) Pattern representing single-cell gene expression of CHRM3 in GBMs. Differential colors indicate the expression levels of individual cells. (C) Violin plot showed the expression levels of CHRM3 in recurrent and primary GBM. (D) Survival curves of patients suffered GBM with low or high expression of CHRM3 in TCGA database, *p* = 0.023. (E, F) Levels of CHRM3 protein (E) and relative quantification (F) in GBM tissues (n = 8) or normal brain tissues (n = 4), *p* = 0.0136. (G) Representative images showed low or high levels of CHRM3 in GBM samples using immunostaining analysis. (H) Survival curves of GBM patients with high or low levels of CHRM3, *p* = 0.002.

After single-nucleus sequencing, t-distributed stochastic neighbor embedding algorithm divided the cells into 19 clusters. CHRM3 was expressed in most of the clusters and was significantly higher in the recurrent group (Figs. 1A–1C and Suppl. Fig. S1). To clarify the prognostic significance of CHRM3 in GBM, survival analysis was performed in TCGA database, which revealed that higher levels of CHRM3 were significantly related to poor survival in GBM patients (Fig. 1D).

To further confirm the importance of CHRM3 in GBM prognosis, the levels of CHRM3 in collected normal brain (n = 4) and GBM tissues (n = 8) were examined using RT-PCR and WB. The WB results showed that compared with normal tissues, CHRM3 was significantly upregulated in the GBM tissues (Figs. 1E and 1F). Furthermore, we detected CHRM3 levels in primary GBM tissue microarrays. Representative staining patterns of CHRM3 in GBM were shown in Fig. 1G. The primary GBM samples were then separated into high (n = 41) and low (n = 24) CHRM3 level groups. Log-rank tests and Kaplan Meier analyses indicated that the patients with high CHRM3 levels displayed significantly worse prognosis than those with low CHRM3 levels (n = 65, *p* = 0.002; Fig. 1H).

Altogether, these data indicated that CHRM3 was significantly upregulated in GBM and predicted poor prognosis not only in TCGA database but also in our GBM samples.

### Knockdown of CHRM3 inhibits GBM cell growth and invasion abilities

**FIGURE 2:**
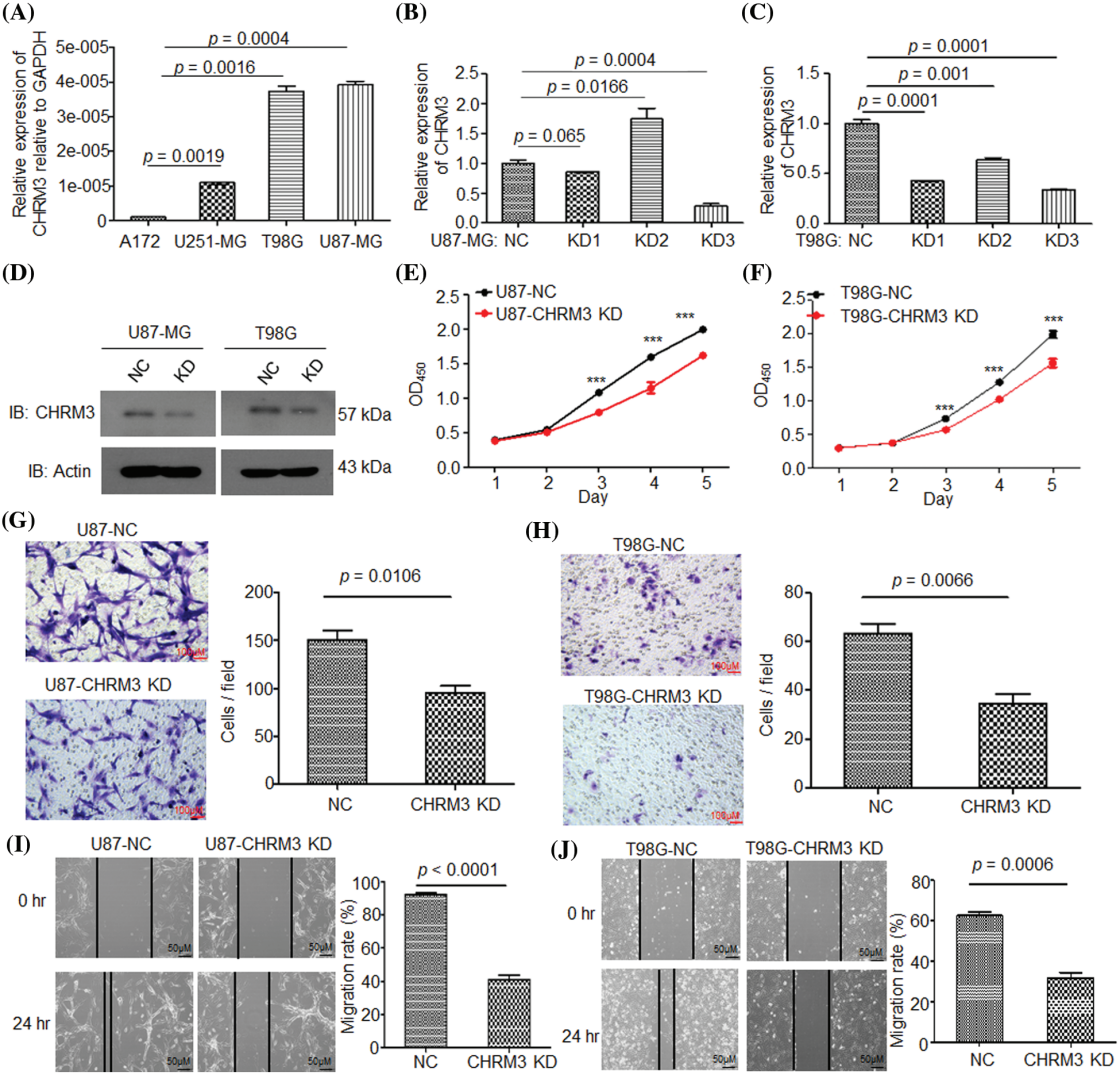
Knockdown of CHRM3 inhibits GBM cell growth and invasion abilities. (A) Relative levels of CHRM3 mRNA in U87-MG, A172, T98G, and U251-MG cells. (B–D) Relative levels of CHRM3 in U87-MG (B, D) and T98G (C, D) with or without CHRM3 knockdown. (E, F) Proliferation abilities of U87-MG (E) and T98G (F) with or without CHRM3 knockdown, ***p < 0.001. (G, H) The numbers of invading cells in U87-MG (G) and T98G (H) with CHMR3 inhibition or negative control. (I, J) Relative migration rate of U87-MG (I) and T98G (J) after scratch with CHRM3 inhibition or negative control. NC = negative control, KD = knockdown.

To clarify the pathological roles of CHRM3 in GBM development, we firstly studied the levels of CHRM3 in an array of cell lines, RT-PCR and WB results displayed that compared to A172 and U251-MG cells, the levels of CHRM3 were relatively higher in U87-MG and T98G (Fig. 2A). We then knocked down the expression of CHRM3 in U87-MG and T98G efficiently, and we found that KD3 is the most efficient sequence. Specifically, 66% and 72% knockdown efficiency of CHRM3 were achieved in T98G and U87 cells, respectively (Figs. 2B–2D).

The pathological roles of CHRM3 in proliferation and invasion abilities of GBM were assessed with or without CHRM3 knockdown. CCK8 showed that the inhibition of CHRM3 significantly reduced cell proliferation abilities, which was significant on the third to fifth days (Figs. 2E and 2F). The Matrigel-transwell invasion assay and scratch assay showed that the inhibition of CHRM3 significantly abridged the invasive capability of GBM cells (Figs. 2G–2J).

### CHRM3 overexpression improves GBM cells growth and invasion abilities

**FIGURE 3:**
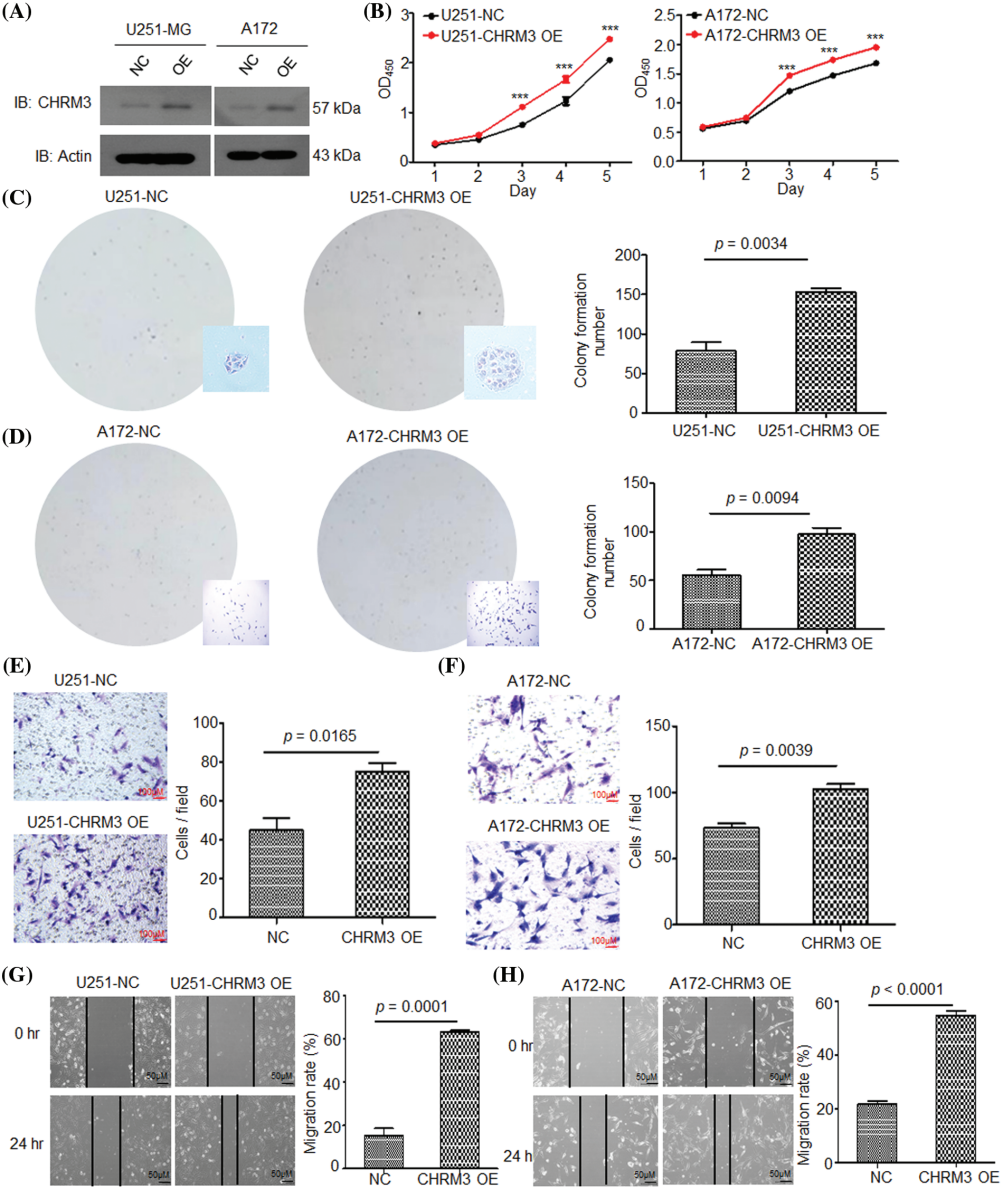
High expression of CHRM3 improves GBM cell growth and invasion abilities. (A) Relative levels of CHRM3 protein in U251-MG and A172 with CHRM3 overexpression or negative control. (B) Proliferation abilities of U251-MG and A172 with CHMR3 overexpression or control cells, ***p < 0.001. (C, D) Number of formed colonies of U251-MG cells (C) and A172 cells (D) with CHRM3 overexpression or negative control. (E, F) The numbers of invading cells on the bottom surface in random fields of transwell assay inserts in U251 (E) and A172 (F) with or without CHMR3 high expression. (G, H) Relative migration rate of U251 (G) and A172 (H) after scrapping with or without CHRM3 knockdown, *p* < 0.0001. NC = negative control, OE = overexpression.

To confirm the pathological role of CHRM3 in GBM growth, we overexpressed CHRM3 in A172 and U251-MG cells. The WB results verified the success of CHRM3 overexpression in these cells (Fig. 3A). Both the CCK8 and colony formation assay showed that the high expression of CHRM3 significantly enhanced cell proliferation abilities (Figs. 3B–3D). The Matrigel-transwell and scratch assay showed that the overexpression of CHRM3 promoted the invasion of GBM cells (Figs. 3E–3H). These findings suggested that CHRM3 was important for the invasive growth of GBM cells.

### Inhibition of CHRM3 suppresses GBM progression and prolongs survival time in animal models

**FIGURE 4:**
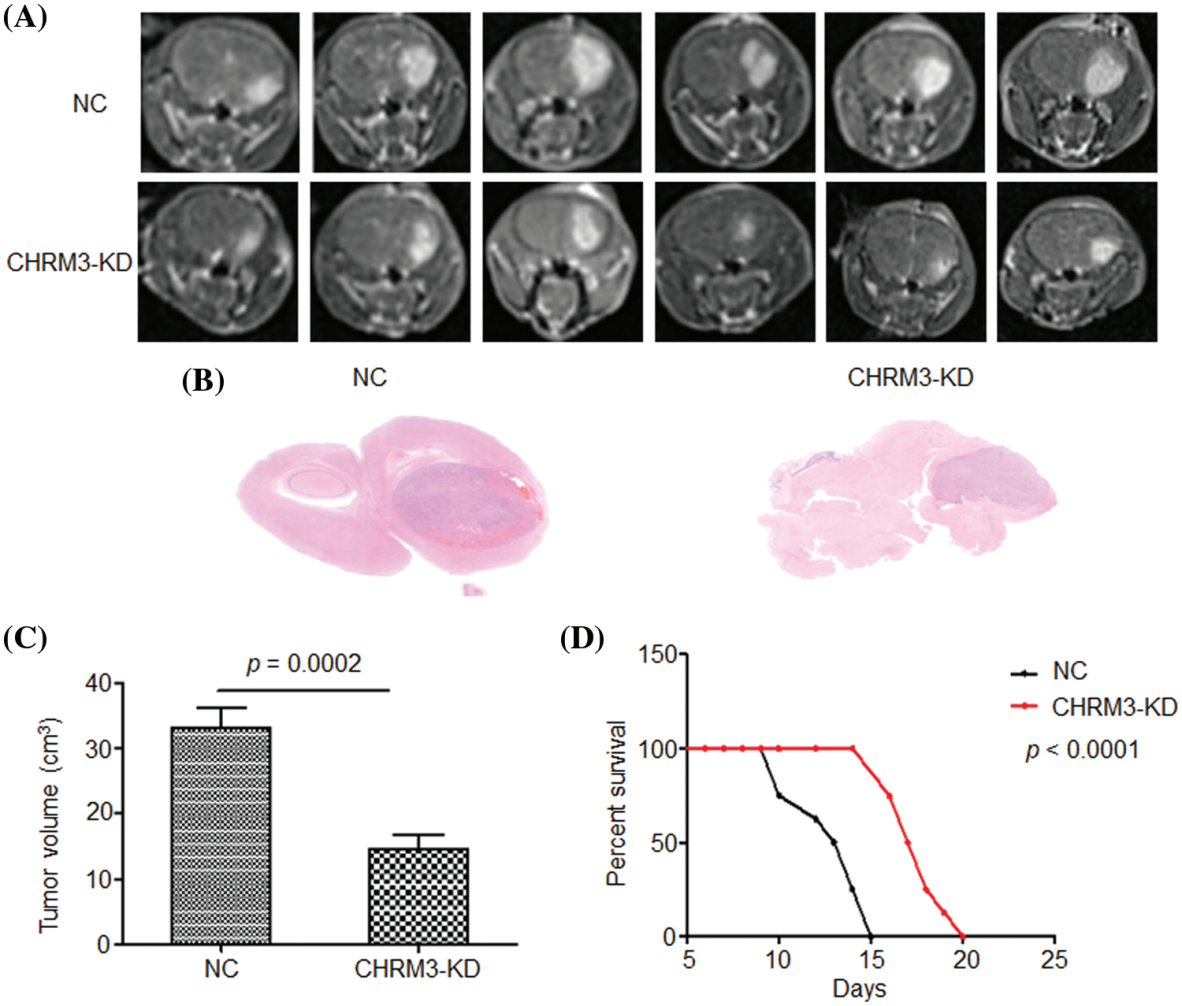
Inhibition of CHRM3 suppresses glioma progression and prolongs survival time in animal models. (A) U87-MG cells were injected into the *corpus* striatum of nude mice (n = 12) with or without CHRM3 knockdown. (B) H&E staining of intracranial tissues of the nude mice with or without CHRM3 inhibition. (C) Tumor volume of U87-MG cells with CHRM3 knockdown or negative control was monitored; *p* = 0.0002. (D) Survival curves of nude mice with or without CHRM3 knockdown; *p* < 0.0001. NC = negative control, OE = overexpression.

To determine the effect of CHRM3 in GBM *in vivo*, we injected U87-MG control cells and CHRM3 knockdown cells subcutaneously into nude mice. The mice with CHRM3-KD exhibited significantly decelerated tumor progression (Figs. 4A–4C). Moreover, the survival of the CHRM3-knockdown mice was prolonged compared with that in the negative control group (Fig. 4D).

### CHRM3 knockdown significantly reduces an array of classic factors in cancer invasive growth

**FIGURE 5:**
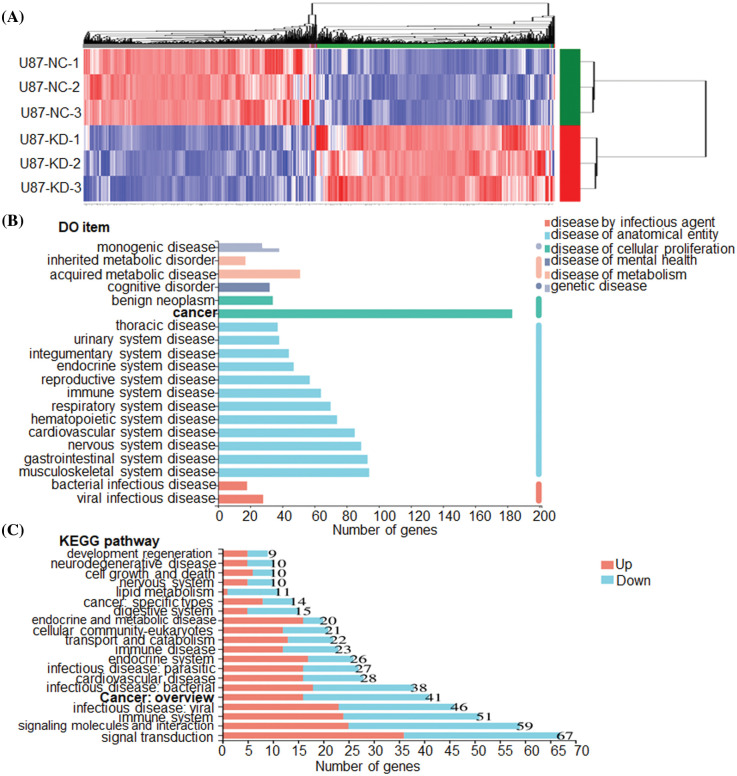
CHRM3 knockdown significantly reduces an array of classic factors involved in cancer. (A) Transcriptome sequencing of U87-MG cells with CHRM3 knockdown or negative control. (B) DO annotations analysis of differentially expressed genes in U87 cells with or without CHRM3 inhibition. Different disease types were presented by different colors. (C) KEGG analysis of differentially expressed genes in U87 cells with or without CHRM3 knockdown.

**FIGURE 6:**
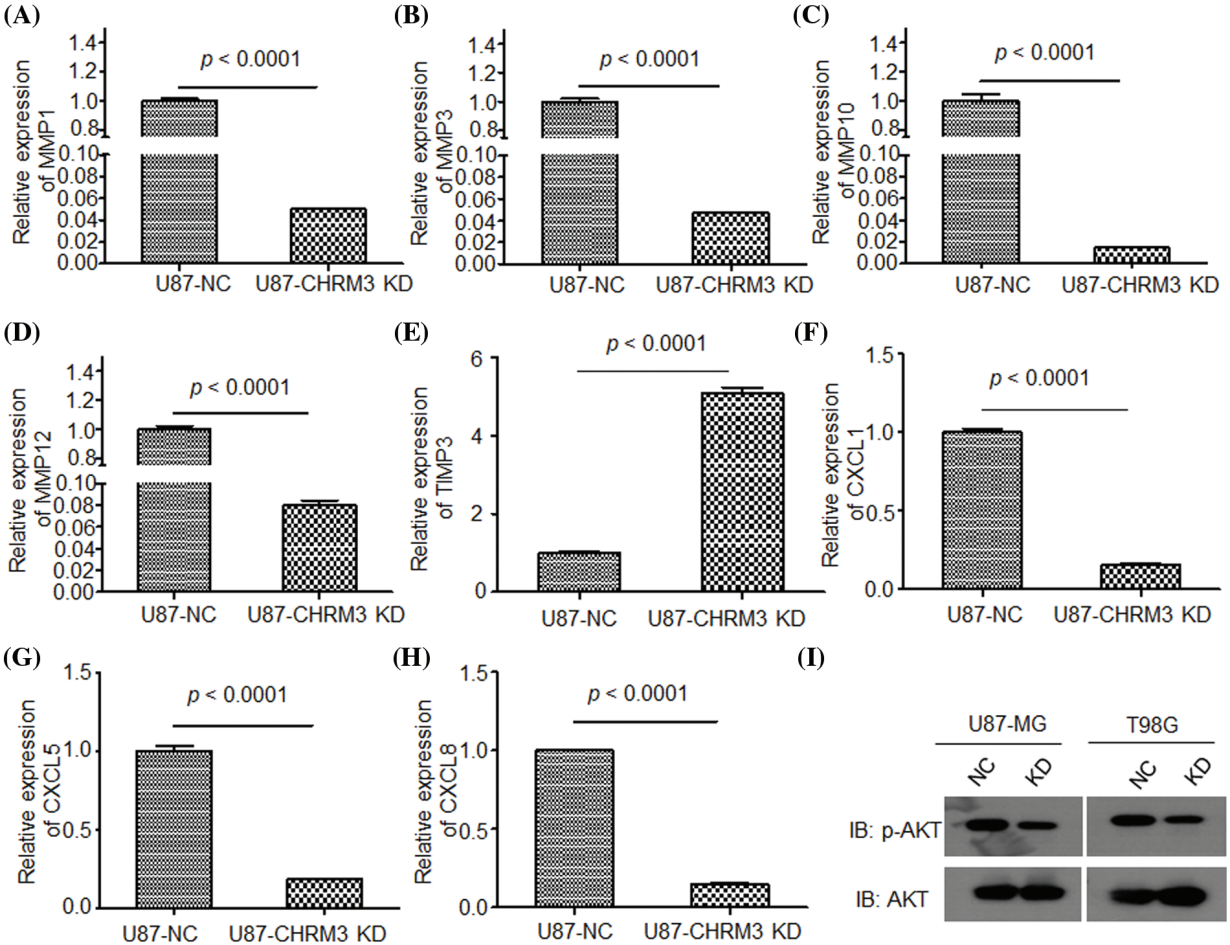
Suppression of CHRM3 down regulate multiple factors involved in cancer invasive growth. (A–H) Relative expression of MMP1 (A), MMP3 (B), MMP10 (C), MMP12 (D), TMP3 (E), CXCL1 (F), CXCL5 (G), CXCL8 (H) in U87 cells with CHRM3 inhibition or negative control; *p* < 0.0001. (I) Levels of p-AKT protein in U87-MG and T98G with or without CHRM3 inhibition.

To clarify the molecular mechanisms of CHRM3 in GBM, transcriptome sequencing was carried out in U87-MG cells with or without CHRM3 knockdown. Transcriptome analysis revealed nearly 600 differentially expressed genes (Fig. 5A). KEGG study and DO annotations analyses showed that most of them are associated with cancer-associated pathways (Figs. 5B and 5C). We then selected seven differentially expressed genes involved in cancer invasive growth for RT-PCR. Consistently, MMP1/MMP3/MMP10/MMP12 and CXCL1/CXCL5/CXCL8 were significantly reduced after CHRM3 knockdown (Figs. 6A–6H). The WB results indicated that p-AKT activation was decreased after CHRM3 suppression (Fig. 6I).

**FIGURE 7:**
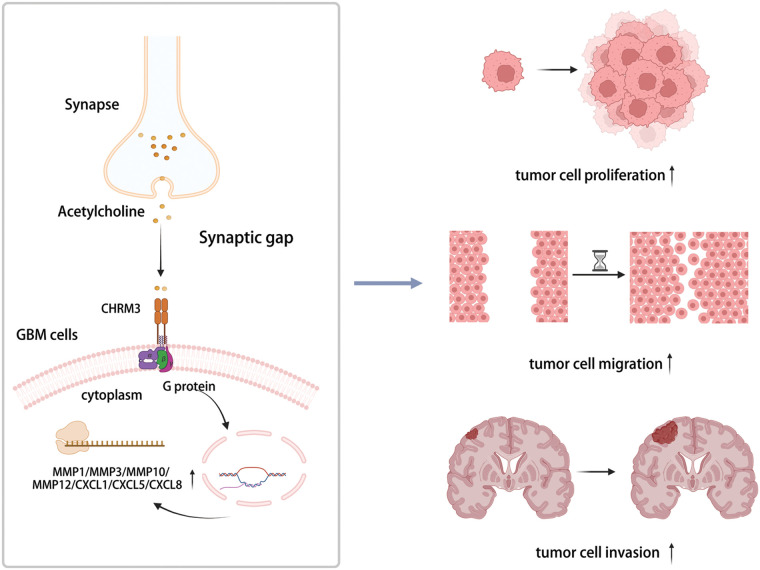
Highly expressed CHRM3 promotes an array of classic factors in cancer invasive growth to promote proliferation and invasion of GBM cells (Created with BioRender.com).

Hence, our data exhibited that CHRM3 levels were significantly amplified in GBM and were related to poor prognosis of GBM patients. Moreover, CHRM3 stimulated GBM development by upregulating an array of classic factors in cancer cells invasive growth (Fig. 7).

## Discussion

The highly infiltrative growth ability of GBM restrains the efficacy of clinical therapy. New prognostic markers and targets are urgently needed to improve GBM treatment. However, the molecular mechanisms of GBM invasive growth stay mostly unknown. In this study, we revealed that the levels of CHRM3 were significantly higher in recurrent samples than in the primary samples, and high expression of CHRM3 predicted poor prognosis of GBM, indicating that CHRM3 might be a new GBM therapeutic target.

Furthermore, our work exposed the pathological roles of CHRM3 in GBM *in vitro* and *in vivo*. Knockdown of CHRM3 reduced the abilities of GBM proliferation, migration and invasion, while overexpression of CHRM3 promoted GBM growth and invasion. As recently reported, CHRM3 and post-M3R signaling activation promote cell proliferation via the EGFR/ERK and MAPK pathways in colorectal cancer [21]. Moreover, CHRM3 activation significantly promotes colorectal cancer invasion via enhanced cellular release of MMP1 [21]. Consistently, we found that CHRM3 was important for expression of an array of MMPs, including MMP1, MMP3, MMP10 and MMP12. Besides, we found that CXCL1, CXCL5, and CXCL8 were regulated by CHRM3, which might be a novel molecular mechanism of CHRM3 in GBM. The CXCL family contributes to immunosuppressive microenvironment in GBM and targeting CXCLs may improve GBM prognosis [25].

However, specific regulation mechanisms between CHRM3 and those classic factors involved in cancer invasive growth in GBM cells remain unclear. Transcription factor regulation after CHRM3 and post-M3R signaling activation needs further studies.

In conclusion, CHRM3 is a new and important factor of GBM progression via regulation of multiple oncogenic genes and these results provide a novel biomarker/target for GBM prognosis and therapy.

## Supplementary Materials

**Figure S1:**
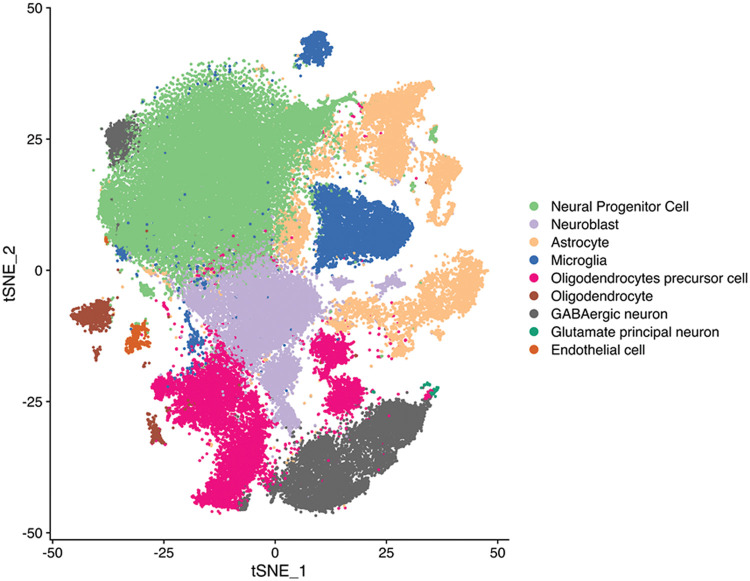
tSEN plot of cells from primary (n = 3) or recurrent (n = 3) GBM patients. Colors denote main cell types.

## Data Availability

All data are available from the corresponding author upon reasonable requests. The single nuclear-sequences data has been deposited in the Genome Sequence Archive in National Genomics Data Center, China National Center for Bioinformation/Beijing Institute of Genomics, Chinese Academy of Sciences (GSA-Human: HRA003631) that are publicly accessible at https://ngdc.cncb.ac.cn/gsa-human. The data discussed in this publication have been deposited in NCBI’S Gene Expression Omnibus [26] and are accessible through GEO Series accession number GSE220083 (https://www.ncbi.nlm.nih.gov/geo/query/acc.cgi?acc=GSE220083).
